# How Readable Are Consultant Psychiatrist Letters From the Mental Health Liaison Team?

**DOI:** 10.1192/bjo.2022.438

**Published:** 2022-06-20

**Authors:** Adam Gadhvi, Katharine McMillan

**Affiliations:** Wye Valley Trust, Hereford, United Kingdom

## Abstract

**Aims:**

To assess whether consultant discharge letters from the mental health liaison team are: 1. Written to patients as advised by NICE shared decision making guidance. 2. Easy to read using the Flesch Reading Ease Test as advised by the Academy of Medical Royal Colleges, which equates to a score of 60 to 70.

**Methods:**

50 consultant discharge letters were collated from April to November 2021. Each letter was assessed whether they were written directly to a patient and scored according to their Flesch Reading Ease (FRE) and Flesch-Kincaid Grade Level (FKGL) via Microsoft Word.

FRE scores a text from 0 to 100 from the average length of sentences and the number of syllables in words to indicate its difficulty to read. The higher the score achieved, the easier it is to read the text. It is a recommended tool by The Academy of Medical Royal Colleges’ guidance on outpatient clinic letters, however, does not specify a target level of readability. A score of 60 to 70 equates to plain English easily understood by students aged 13 to 15 years and was concluded to be the equivocal score expressed in the guidance.^4^

The FKGL presents a score as a U.S. grade level to indicate the level of education generally required to understand a text. Words per sentence and syllables per word are factored in to calculate the grade.^5^

**Results:**

The median FRE was 50.9 (n = 50, IQR 8.9). Only one letter met the desired standard. The mean score was 50.6 (SD 6.4). This mean was significantly different from a hypothetical ideal mean of 65 (t(df) = 15.9(49), p < 0.0001) so could not, unfortunately, be explained by chance. The median FKGL was 10.1.

**Conclusion:**

Overall, the letters were of greater difficulty than the desired score of both FRE and FKGL. Lay language and patient-directed writing will aid in improving scores.

